# Orthopaedic knee scooter-related injury: prevalence and patient safety perception in a prospective cohort with exploratory risk factor analysis

**DOI:** 10.1186/s13018-023-04124-6

**Published:** 2023-09-02

**Authors:** John P. Walsh, Mark S. Hsiao, Landon Rosevear, Ryland McDermott, Shivali Gupta, Troy S. Watson

**Affiliations:** 1 Department of Orthopaedic Surgery, Valley Hospital Medical Center, 620 Shadow Lane, Suite 450, Las Vegas, NV 89121 USA; 2 The Foot and Ankle Institute at Desert Orthopaedic Center, Las Vegas, NV USA; 3 Kirk Kerkorian School of Medicine at UNLV, Las Vegas, NV USA

**Keywords:** Injury, Foot and ankle surgery, Orthopaedic knee scooter (OKS), Orthopedic equipment, Surveys and questionnaires, Prospective study

## Abstract

**Background:**

There is a paucity of research investigating the harms associated with orthopaedic knee scooter (OKS) use and patient safety perceptions. This prospective study aimed to define the prevalence of OKS-related injuries, describe the patient perceptions of OKS safety, and identify potential risk factors.

**Methods:**

This study was conducted at a single foot and ankle fellowship-trained surgeon’s community-based clinic from 6/2020 to 4/2021 and enrolled 134 patients. Our primary outcome was an OKS-related event (injury or fall) and informed an a priori power analysis. Point estimate of association magnitude was calculated as an odds ratio (OR) for statistically and clinically significant associations.

**Results:**

There were 118 (88%) patients eligible for analysis; fourteen enrolled patients did not use OKS, and two withdrew. The prevalence of patient falls was 37% (44/118), and the prevalence of patient injury was 15% (18/118). Four percent of patients would not recommend OKS and 8% would not use an OKS again. Sedentary lifestyle increased risk (OR = 4.67, 1.52–14.35 95 CI) for OKS-related injury.

**Conclusions:**

Despite a high prevalence of patient falls (37%), there is a low prevalence of injury (15%) and a favorable perception of OKS safety. Sedentary lifestyles may be a risk factor for OKS-related injury and should be considered in the development of a risk model.

**Supplementary Information:**

The online version contains supplementary material available at 10.1186/s13018-023-04124-6.

## Background

The orthopaedic knee scooter (OKS) is an alternative mobility aid to canes, crutches, and walkers to facilitate nonweightbearing (NWB) patients after lower extremity injury or surgery. Reported advantages of the OKS include less energy expenditure, lower perceived exertion, and faster walking velocity when compared to crutches [1–3]. Further, patient satisfaction is high and preferred by many patients. One prospective crossover study compared an OKS to axillary crutches, showing that 88% of patients preferred utilizing an OKS over crutches [3]. Additionally, a retrospective descriptive study analyzed patient satisfaction with the use of an OKS where 85% of patients were satisfied compared to 13% that were dissatisfied [4].

Healthcare providers have sought to reduce perioperative risks, and the risk for patients undergoing foot and ankle surgery is uniquely prolonged due to routine immobilization and prolonged NWB restrictions [5–7]. Only two reports have investigated OKS safety: one retrospective study reported ≥ 40% of patients experienced a fall, and another survey of surgeon members in the American Orthopaedic Foot and Ankle Society estimated a 2.5% prevalence of scooter-related injuries [4, 8]. Thus, there exists a paucity of literature regarding OKS safety and may be a discrepancy between the OKS safety perceptions between patients and surgeons. Defining the prevalence of events (fall or injury) may help inform the magnitude of complications secondary to OKS-related events.

The primary purpose of the study was to describe and quantify the prevalence of OKS-related injuries in a cohort followed longitudinally. Clinical experience has informed our supposition that there are many falls leading to injuries experienced by patients that are underestimated and incompletely understood. Secondary purposes included describing patient perceptions regarding OKSs and analyzing patient characteristics for associations with OKS-related injuries to identify potential risk factors. We hypothesize patient characteristics, specifically age and weight, may influence the risk of sustaining an OKS-related injury. This may provide actionable information that is quantifiable for data-driven preventive interventions by healthcare providers, thus reducing patients’ complication burden. Our secondary hypothesis is that patients have an overall positive perception of knee scooters due to the benefits they provide over the traditional crutches and wheelchair.

## Methods

This prospective study was conducted at X.X.X. in Z.Z.Z., a community-based orthopaedic clinic. All patients for this observational study were selectively recruited at a single foot and ankle fellowship-trained surgeon’s clinic from 6/2020 to 4/2021. Inclusion criteria for this study: (1) undergoing surgery with the senior surgeon (Y.Y.Y), (2) required NWB period postoperatively, (3) planned to use OKS to facilitate NWB and (4) agreed to complete all three surveys. Exclusion criteria included: (1) non-English speaker and (2) patients unable to complete electronic surveys independently. Patients that provided consent completed an Enrollment Survey preoperatively and were followed longitudinally during their NWB period postoperatively. Options for mobility devices (crutches, OKS, wheelchair) were thoroughly discussed preoperatively and postoperatively, and the different options were physically shown to the patients in the clinic. Patients were free to choose their desired device. Patients were given general training in the clinic on how to use their selected device based on manufacturing instructions without any specific evidence-based training or recommendations due to limited studies in the literature. Patient information was collected through an electronic survey, de-identified and entered in an encrypted and password-protected electronic database in a HIPAA compliant G suite (Alphabet, Mountainview, California, USA) account. This research project received an administrative review and was determined to be exempt from full board review by the Touro University Nevada Institutional Review Board (IRB) (IRB#:TUNIRB000084). This research qualified for exemption under Category 2. Category 2 entails human interactions involving educational tests, such as survey studies, with limited risk to participants.

### Outcomes measures

The surveys aimed to document OKS-related events and gauge patient perceptions (Additional file 1). Patient data was collected using three electronic surveys administered during clinic visits using standardized data collection protocols. Demographic data, including sex, age, height, weight, diabetes (yes/no), were collected using the Enrollment Survey (Survey #1). Scooter Event Surveys (Survey #2) were administered electronically to enrolled patients while in the clinic exam room. The Enrollment Survey (Survey #2) also assessed patient perceptions of OKS safety using a Likert Scale (1–5), with 1 defined as “not safe at all” and 5 defined as “completely safe.” The Exit Survey (Survey #3) was completed after weightbearing restrictions were removed. All three surveys can be found in Additional file 1: Appendix 1. Patient perceptions regarding the OKS and future behaviors were queried in the Exit Survey, including a repeat assessment of perceived safety. Survey questions were dichotomized (yes/no) for analysis. The participants completed their surveys electronically, and responses were kept anonymous. The survey is not a validated questionnaire of measure of any outcome. Considering that several the date of NWB restriction removal was not always concordant with clinic appointment dates or compliant with the senior surgeon’s recommendations, there was significant uncertainty surrounding specific time at risk and not included as a dimension of analysis.

### Statistical analysis

**Fig. 1:**
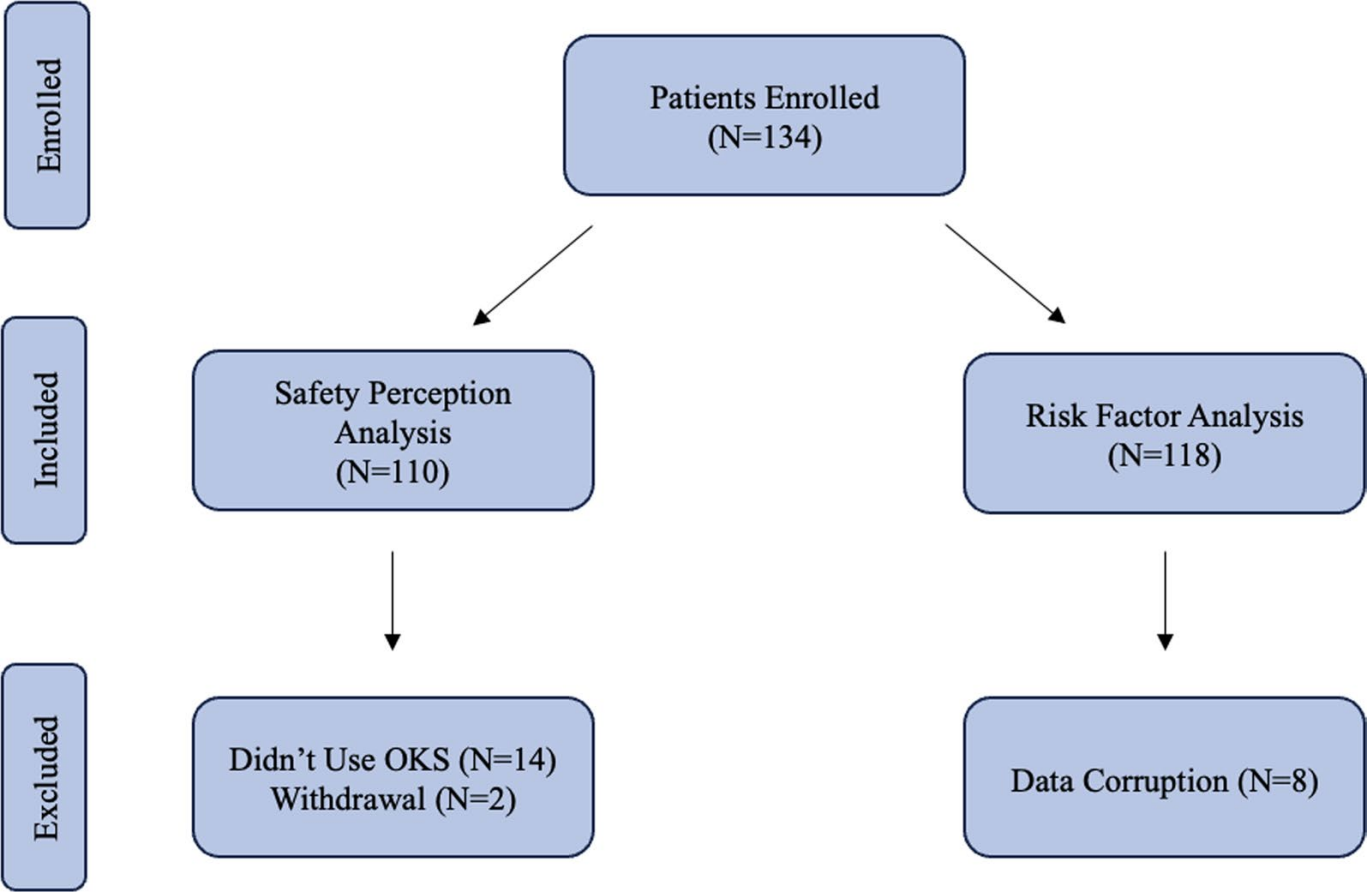
Patient flow chart

We planned a prospective study of independent cases. Our primary outcome, defined an event (injury or fall), and was estimated from a previously reported prevalence of 0.025 [8]. If the standardized difference of event rate for experimental subjects is 0.20, we will need to study 104 subjects to reject the null hypothesis that the event rates for experimental and control subjects are equal with probability (power) 0.8. The Type I error probability associated with this test of this null hypothesis is 0.05. We will use a Fisher Exact and Chi-Square Test to evaluate this null hypothesis. A priori power analysis was conducted using G*Power (Universitat Dusseldorf, Dusseldorf, Germany) [9]. To accommodate uncertainty in estimation, survey data, and attrition, our target enrollment was inflated 30% to 134 patients. Between 6/2020 and 4/2021, 134 patients consented to participate in the study. See Fig. 1 below for the patient flow chart.

The primary outcome was the prevalence of patients that experience an event (fall or injury), reported with descriptive statistics. For this study, clinical significance was defined as a patient that experienced an OKS-related fall that resulted in injury. We describe patient perceptions using an Ordinal Likert Scale. Secondary exploratory analysis of patient variables assessed for associations to identify potential risk factors. Continuous variables were analyzed using nonparametric tests. A comparison of categorical values was performed using the Fisher Exact test. For statistically and clinically significant associations, a point estimate of the association magnitude was calculated as an odds ratio (OR) according to following appropriate use guidelines [10, 11]. Alpha *P* < 0.05 was considered statistically significant, and 95% confidence intervals were reported (95 CI). Exploratory analyses were conducted, so multiple testing adjustments were not made. Statistical analysis was performed using MedCalc (MedCalc Software, Ostend, Belgium). Figures and tables were generated using Microsoft Office (Microsoft Inc., Redman, Washington, USA) and R (R Foundation for Statistical Computing, Vienna, Austria) [12].

## Results

The cohort included 118 patients with an average age of 48 years (range 10–74) and was 60% (*N* = 71) female as seen below in Table 1. Fourteen enrolled patients did not use an OKS and two patients withdrew. Five patients (4.2%) reported more than one fall, of which four (80%) also reported an OKS-related injury. No patient reported more than one injury.

**Table 1:** Cohort composition

Variables	Cohort (N = 118)	Injured (18)	Uninjured (100)	*P*-Value
Gender: *N* (%)				1
Male	47 (40)	7 (39)	40 (40)	
Diabetes: *N* (%)				0.63
Yes	9 (8)	2 (1)	7 (7)	
Lifestyle: *N* (%)				0.01
Active	99 (84)	11 (61)	88 (88)	
Sedentary	19 (16)	7 (39)	12 (12)	
Age (years): avg (range)	48 (10–74)	55 (28–74)	47 (10–73)	0.08
Height (in): avg (range)	67 (61–77)	67 (63–76)	68 (61–77)	0.19
Weight (lbs): avg (range)	189 (110–330)	187 (135–290)	200 (110–330)	0.35

Years (yrs); average (avg); standard deviation (SD); inches (in); pounds (lbs)

The prevalence of patient falls was 37% (44/118, 95 CI 29–46%), and the prevalence of patient injury was 15% (18/118, 95 CI 10–23%). Of the injured patients, 94% (17/18) reported a prolonged recovery secondary to their injury. The prolonged recovery reported averaged 7 (range: 5–10) weeks. Twelve of eighteen (67%) injured patients required evaluation by a healthcare provider, including radiographic imaging (4/18, 33%) or an MRI (1/18, 8%). No patient in this study required a second surgery because of their injury.

Injured patients had significantly lower safety perceptions at study exit (*P* < 0.01) compared to uninjured patients but were not significantly different from their perceptions at enrollment as seen in Table 2 below. Safety perceptions did not significantly differ between time points.

**Table 2:** Patient perceptions of orthopaedic knee scooter (OKS) safety

Patient safety perception-intragroup	Median (interquartile range)	*P*-value
Total cohort (*N* = 110):		0.81
Enrollment	4 (4–5)	
Exit	4 (4–5)	
Injured (*N* = 17):		0.5
Enrollment	4 (3–5)	
Exit	3 (2.8–4.3)	
Uninjured (*N* = 93):		0.49
Enrollment	4 (4–5)	
Exit	4 (4–5)	
Enrollment		0.05
Uninjured (*N* = 93)	4 (4–5)	
Injured (*N* = 17)	4 (3–5)	
Exit		< 0.01
Uninjured (*N* = 93)	4 (4–5)	
Injured (*N* = 17)	4 (4–5)	

Four percent (4/110) of patients would not recommend using an OKS to a friend as outlined in Table 3. There were 8% (9/110) of patients who would not use an OKS in the future, and there was a significant difference between injured and uninjured patients (*P* = 0.03). Three uninjured patients documented a preference for using a wheelchair in the future, and two uninjured patients would use crutches. Data corruption for 7% (8/118) patients precluded inclusion in the analysis.

**Table 3:** Patient behavior and orthopaedic knee scooter (OKS) use

Patient behaviors (*N* = 110)	*N* (%)	Injury	Uninjured	*P*-value
Recommend scooter to friend				0.112
Yes	106 (96)	15	91	
No	4 (4)	2	2	
Use scooter in future: *N* (%)				0.031
Yes	101 (92)	13	88	
No	9 (8)	4	5	

Exploratory analysis revealed that the odds of OKS-related injury are significantly greater (*P* = 0.01) in patients that report a sedentary lifestyle (OR = 4.67, 1.52–14.35 95 CI). Age, height, weight, and diabetes were not associated with OKS-related injury.

## Discussion

The prevalence of OKS-related falls was 37% (44/118), and the prevalence of OKS-related injury was 15% (18/118). Despite the high prevalence of falls, patients tend to perceive OKSs favorably, as only 4% would not recommend an OKS to a friend, and only 8% would not use an OKS in the future. OKS-related injury and the subsequent reduction in the safety perceptions may have future implications on patient behavior as significantly less injured patients would use an OKS in the future (*P* = 0.03). Our exploratory analysis supported our secondary hypothesis that suggested a sedentary lifestyle had significantly increased odds (OR = 4.67, 1.52–14.35 95 CI) of OKS-related injury.

The estimated postoperative prevalence of OKS-related injuries was calculated using a survey of AOFAS members was 2.5% [8]. The authors used a web survey to characterize postoperative OKS-associated injuries, and respondents reported that 34% of OKS-related injuries were treated operatively. Similarly, we reported a 15% prevalence of OKS-related injuries in a prospective cohort, but no injury required secondary surgery. The disparate data may be a function of the infrequent but consequential impact of OKS-related injuries, which may explain why we did not observe the infrequent catastrophic event in our small cohort [13, 14]. The disparate secondary surgery data may be influenced by surgeon/patient cognitive biases, or technical biases, but warrants further investigation due to potential consequential impacts [15–18]. OKS-related injuries necessitating secondary surgery may be infrequent catastrophic events and likely absent in our small cohort [19]. Engaging patients who have experienced an adverse event may help characterize adverse events to provide insight into unmet patient needs that guide selection of mobility aid. One study found that 66% of patients did not receive any instruction about using an OKS, despite the well documented importance of understanding patient needs and functions for appropriate device selection [20–22].

Two studies have reported OKS-related risk factors [4, 8]. One survey of AOFAS members implicated female sex, older age, obesity, and sedentary lifestyle with scooter-related injuries [8]. Our data corroborated that a sedentary lifestyle is a risk factor for OKS-related injury. Patients with a sedentary lifestyle may have impaired mobility or physical ability, predisposing patients to fall with subsequent increased risk of injury. Prior community-based investigation of a sample of elderly (mean age of 83 years) participants found mobility impairment to be the strongest risk factor for patient falls [23]. Importantly, the authors concluded the strongest risk factor is modifiable and amenable to preventive strategies. In our cohort, non-modifiable factors such as sex, age, height, and weight were not identified as potential risk factors, but one modifiable factor (sedentary lifestyle) may be a specific risk factor for clinically significant events (OKS-related injury) and provide a practical target for preventive interventions. One retrospective review found no association between OKS-related falls and gender, age, BMI, or the number of comorbidities [4]. Our analysis differed from the prior report as we focused on OKS-related injury, but we corroborated their findings and did not find age or sex as risk factors for OKS-related events (fall or injury). Both cohorts had a similar demographics and frequency of patient falls (37% and 44%). Nevertheless, multiple testing and sparse event data generate spurious findings and experimental studies are required to support preliminary observational data [24, 25].

Only one study has reported patient falls and satisfaction when using an OKS [4]. Their retrospective review of patient survey data revealed that 43% of patients reported a fall when using an OKS postoperatively. The authors concluded that patient satisfaction after using an OKS is high. Our samples were similar, predominantly female with a mean age within the 50 s. Patients that reported a fall in our sample were slightly lower at 37%. Our data echoed their patient satisfaction (85% patient satisfaction) findings as 92% of our sample would use an OKS in the future. Their survey asked about patient perceptions after using an OKS, and we inquired about their perception before and after, which dynamically captured the expected reduction in perceptions of safety and quantified the OKS-injury impact.

All patients would benefit from OKS education but identifying at-risk patients may help reduce events that result in injury. The importance of understanding patient needs and functions for appropriate device selection is well documented [20–22]. Our data enables speculation that OKS-related falls do not impact patient perceptions or behavior, though our study was not powered to detect this outcome and is susceptible to type II error. Despite sustaining an injury and reduced perception of safety, only 3.4% of patients in our sample would not recommend an OKS to a friend. Patient safety perceptions decreased in injured patients, though the magnitude did not appear to influence future behavior and should be further investigated. This study could be of interest to OKS engineers who can modify the assembly to improve patient acceptance and safety. Thus, the mechanism of injury should also be further investigated for OKS manufacturers. One study found that 66% of patients did not receive any instruction about using an OKS [4]. Engaging patients that have experienced an adverse event may help characterize adverse events to provide insight into unmet patient needs that guide mobility aid selection [20].

Our data intended to document the patient perception with OKSs to inform the development of patient-oriented education or preventive interventions to reduce OKS-related injury. Preventive intervention like the introduction of regular exercise regimens to older adults was found to prevent fall-related fractures, with a 0.74 RR calculated in a meta-analysis of randomized controlled trials [26]. Non-modifiable risk factors (such as sex or age) do not appear to influence the risk of injury. In the setting of elective surgery, patient education may reduce the risk of sedentary lifestyles and presents an opportunity for providers to reduce perioperative risks. Future studies should aim to assess the effectiveness of patient education, provider screening measures, or preventive interventions in reducing OKS-related injury.

There are several strengths to this study. First, our data highlighted the common occurrence of OKS-related events and provided providers with actionable information with simple intervention (screening questions or education materials). Second, performance bias was reduced (but not eliminated) by following standardized data collection, electronic data collection, and storage. Third, detection bias was mitigated by using a prospective study design and proactive patient questioning. Recall bias was mitigated through repeated survey administration. Fourth, our study addresses a deficiency in the literature and may precipitate further OKS research. Fifth, an a priori power analysis was conducted to ensure the study was adequately powered. Sixth, prospective studies provide more valid rate and risk estimates contingent on temporality certainty. Lastly, STROBE and SAMPL reporting guidelines were referenced during study design and manuscript preparation to ensure methodologic rigor and transparency [27, 28].

Limitations of this study include information bias. Data are dependent on patient reporting and limited by patient recall and definition. We believe the number of falls is likely underreported. Further, the distinction between a fall and injury is likely varied among patients and influenced by the event consequences. Considering the likely impact on patient perception, the authors believe that the number of injuries is likely more accurately reported than falls and that most clinically significant events would be reported as an injury. Second, the absolute event rates were low, reducing the precision of our analysis and may be influenced by sparse-data bias [18, 25]. More event data is required and may be achieved with a multicenter investigation. Our data provided prospective, patient-reported event rates to inform multicenter study enrollment or baseline literature comparisons. Third, the cohort was treated by a single surgeon in a foot and ankle specialty clinic; therefore, the results of this study may have limited external validity. Additionally, the study only included patients that underwent surgery, creating sampling bias and potentially further limiting external validity. Future studies can include different national and/or international patient populations as well as patients who sustained non-operative foot and ankle injuries that required immobilization to improve the generalizability of the results. Importantly, we aimed to document the previously unreported prevalence of OKS-related injuries and identify patient risk factors. Lastly, the current report is restricted by the intrinsic limitations of an observational study. Nevertheless, this report is intended to be exploratory rather than to validate. Given the paucity of data regarding OKS-related injuries and risks, we believe this study adds value to the existing literature.

## Conclusion

Despite a high prevalence of patient falls (37%), there is a low prevalence of injury (15%) and a favorable patient perception of OKS safety. Sedentary lifestyles may be a risk factor for OKS-related injury and may be considered in developing a risk model and assessing the effectiveness of patient education or provider screening measures in reducing OKS-related injury.

### Supplementary Information

**Additional file 1. Section 1:** Enrollment Questionnaire. **Section 2:** Knee Scooter Questionnaire. **Section 3:** Exit Questionnaire.

## Data Availability

All data generated or analyzed during this study are included in this published article (and its Additional file 1).

## Abbreviations

OKS: Orthopaedic knee scooter
OR: Odds ratio
NWB: Nonweightbearing
