# Supplementary material for: Orthopaedic knee scooter-related injury: prevalence and patient safety perception in a prospective cohort with exploratory risk factor analysis
Source: J Orthop Surg Res. 2023 Sep 2;18:649. doi: 10.1186/s13018-023-04124-6 (PMC10474665; doi:10.1186/s13018-023-04124-6)
Supplement: Supplementary file 1 — Additional file 1. Section 1: Enrollment Questionnaire. Section 2: Knee Scooter Questionnaire. Section 3: Exit Questionnaire. [file 13018_2023_4124_MOESM1_ESM.docx]

**Section 1: Enrollment Questionnaire**

Please select how you identify:

- Male
- Female

Please enter your age (years): ____

Please enter your height (ft): ____

Please enter your weight (lbs): ____

Do you have diabetes?

- No
- Yes

How would you describe your lifestyle?

- Sedentary
- Active

Do you feel a knee scooter is a safe form of mobility?

- 1 (Not Safe At All)
- 2
- 3
- 4
- 5 (Completely Safe)

**Section 2: Knee Scooter Questionnaire**

Please enter your unique patient identifying number: ____

Did you have a scooter-related fall?

- Yes
- No

Did you sustain a scooter-related injury secondary to an accident, fall, or otherwise related to your scooter?

- Yes
- No

If yes to injury, did you require medical evaluation?

- Yes
- No

If yes to injury, did you require diagnostic imaging?

- Yes
- No

If yes to diagnostic imaging, what diagnostic imaging was required?

- X-ray
- MRI
- CT scan
- No additional imaging required

Did the injury necessitate a second surgery?

- Yes
- No

What were the secondary procedures performed? ____ (Leave blank if unknown)

Did the injury prolong your postoperative recovery period?

- Yes
- No

How many weeks longer was your recovery period? ____ weeks

**Section 3: Exit Questionnaire**

Do you feel a knee scooter is a safe form of mobility?

- 1 (Not safe at all)
- 2
- 3
- 4
- 5 (completely safe)

Would you recommend a knee scooter to a friend in the future?

- Yes
- No

Would you choose to use a knee scooter, crutches, or wheelchair for a future foot surgery?

- Knee Scooter
- Crutches
- Wheelchair
